# The impact of an orthogeriatric intervention in patients with fragility fractures: a cohort study

**DOI:** 10.1186/s12877-019-1299-4

**Published:** 2019-10-15

**Authors:** Charlotte Abrahamsen, Birgitte Nørgaard, Eva Draborg, Morten Frost Nielsen

**Affiliations:** 1 Department of Orthopaedic Surgery, Kolding Hospital a part of Hospital Lillebaelt, Kolding, Denmark; 2 Department of Public Health, University of Southern Denmark, Odense, Denmark; 3 Endocrine Research Unit & KMEB, Odense University hospital, Odense, Denmark

**Keywords:** Frail elderly, Fragility fracture, Osteoporotic fractures, Orthogeriatric, Postoperative complications

## Abstract

**Background:**

While orthogeriatric care to patients with hip fractures is established, the impact of similar intervention in patients with fragility fractures in general is lacking. Therefore, we aimed to assess the impact of an orthogeriatric intervention on postoperative complications and readmissions among patients admitted due to and surgically treated for fragility fractures.

**Methods:**

A prospective observational cohort study with a retrospective control was designed. A new orthogeriatric unit for acute patients of sixty-five years or older with fragility fractures in terms of hip, vertebral or appendicular fractures was opened on March 1, 2014. Patients were excluded if the fracture was cancer-related or caused by high-energy trauma, if the patient was operated on at another hospital, treated conservatively with no operation, or had been readmitted within the last month due to fracture-related complications.

**Results:**

We included 591 patients; 170 in the historical cohort and 421 in the orthogeriatric cohort. No significant differences were found between the two cohorts with regard to the proportion of participants experiencing complications (24.5% versus 28.3%, *p* = 0.36) or readmission within 30 days after discharge (14.1% vs 12.1%, *p* = 0.5). With both cohorts collapsed and adjusting for age, gender and CCI, the odds of having postoperative complications as a hip fracture patient was 4.45, compared to patients with an appendicular fracture (*p* <  0.001). Furthermore, patients with complications during admission were at a higher risk of readmission within 30 days than were patients without complications (22.3% vs 9.5%, *p* <  0.001).

**Conclusions:**

In older patients admitted with fragility fractures, our model of orthogeriatric care showed no significant differences regarding postoperative complications or readmissions compared to the traditional care. However, we found significantly higher odds of having postoperative complications among patients admitted with a hip fracture compared to other fragility fractures. Additionally, our study reveals an increased risk of being readmitted within 30 days for patients with postoperative complications.

## Background

For decades, hip fracture has been the most common fragility fracture among the elderly and a well-known cause of significant health-related challenges in terms of increased mortality and comorbidity, as well as increased health care costs [1, 2]. To address these challenges, orthogeriatric care was developed as a model of collaboration between geriatricians, orthopaedic surgeons, and an interprofessional team of nurses, therapists, and others [3]. Orthogeriatric care has been shown to decrease the prevalence of postoperative complications [4–10], readmission rates [4, 5, 9, 11], and mortality [4, 6, 8, 11–16] in hip fracture patients, as compared to traditional orthopaedic care. The majority of these studies were designed as retrospective [6, 7, 9, 10, 12–14, 16] or prospective cohort studies with a historical cohort [4, 5, 11, 15], and only one study as a randomized controlled trial [8].

Until recently, orthogeriatric care was predominantly delivered to elderly patients with hip fractures; however, fragility fractures in elderly patients may occur in several bones within the appendicular or axial skeleton [17]. Thus, awareness of orthogeriatric care for patients with various fragility fractures is increasing [18, 19].

Only a limited number of investigations of the orthogeriatric care of patients with various fragility fractures have been published including two investigations that did not include comparisons to traditional orthopaedic care were performed [20, 21]. Furthermore, one study reported that, compared to conventional care, admission to wards where physician consultations and multidisciplinary care conferences were available increased the odds of one-year survival after hip or lower extremity injury in elderly patients [22].

Based on a review of the literature, a panel of experts in hip fracture management has recommended that twelve outcome parameters are used in the evaluation of orthogeriatric care—namely, mortality, length of stay, time to surgery, postoperative complications, readmission rate, mobility, quality of life, pain, activities of daily living, medication use, place of residence, and cost [23].

Postoperative medical complications in older hip fracture patients are common and are known to increase the length of stay in hospital and the overall cost of care [24]. Furthermore, these complications impair the patients’ ability to return to their previous functional status and increase mortality [8, 25].

In order to optimize the care pathway for elderly patients with fragility fractures and to gain more knowledge about its effect, we implemented an orthogeriatric care unit.

The primary objective of our study was to assess the impact of an orthogeriatric intervention on postoperative complications in patients admitted due to and surgically treated for fragility fractures. We hypothesized that an orthogeriatric intervention in patients with fragility fractures would decrease the incidence of in-hospital postoperative complications. Secondly, we wanted to assess readmission rates with a notion of reduction.

## Methods

The study concerned a regional hospital with no co-payment, serving a mixed rural and urban district in Denmark. The hospital provided 24-h emergency assessment, orthopaedic surgery and internal medicine services. It furthermore had an ICU. A new orthogeriatric unit for acute patients of sixty-five years or older with various fragility fractures was opened on March 1, 2014. From that date, all patients of sixty-five years or older with fragility fractures in terms of hip and appendicular fractures were transferred directly to the new orthogeriatric unit after examination in the emergency room.

### Intervention

The orthogeriatric unit was staffed by an interprofessional team consisting of orthopaedic surgeons, geriatric specialists, nurses, nursing assistants, physiotherapists, occupational therapists, and dieticians collaborating on the treatment and care of patients with fragility fractures.

Each weekday, an interprofessional conference was conducted, in which treatment, training, nursing care, and discharge planning for each patient was discussed. Furthermore, on weekdays, patients were assessed in ward rounds and receiving daily physiotherapy training. Patients with severe functional challenges were offered training in daily living activities by occupational therapists. Where relevant, plans for early discharge were discussed with the patients and their families. For all patients who had previously received municipal home care, a discharge report was sent to the home care service. If major changes at home were needed, a video conference between patient, relatives, home care, and nurses from the ward was conducted. For further details on the distinction between orthogeriatric care and traditional orthopaedic care, see Table 1.

**Table 1:** Outline of organizational, training, and care path differences between historical and orthogeriatric cohort

Activities	Traditional orthopaedic care		Orthogeriatric care
	Patients with hip fractures	Patients with other fragility fractures	Patients with hip and other fragility fractures
Interprofessional conference	None	None	Interprofessional team meetings every weekday.
Ward round	The geriatrician attended the ward 2 × 1 h per week, reading patient medical records and recommending further medical examination and treatment. The orthopaedic consultant was responsible for patient treatment.	The orthopaedic consultant had the sole responsibility for patient treatment	The geriatrician attended the ward every day Monday to Friday. The geriatrician and orthopaedic consultant shared responsibility for patients. They attended to patients according to medical importance.
Treatment	Routine prescription of calcium and vitamin D and fall prevention, when relevant	No routine prescriptions	Systematic prescription of calcium and vitamin D and fall prevention, when relevant. Systematic orthostatic blood-pressure measurement; routine blood tests concerning medical status.
Follow-up round	None	None	Follow-up rounds each afternoon by the geriatrician and orthopaedic consultant. Follow-up on x-ray, blood tests, subacute matters, etc.
Training facilities in the ward	None	None	A dedicated room with exercise equipment used for group and individual training, Monday to Friday
Physiotherapy	Individual training and evaluating walking aids (mean time 140 min per patient per admission)	Individual training and evaluating walking aids (time not assessed).	Daily individual training and group training and evaluating walking aids (mean time 250 min per hip patient during admission).
Occupational therapy	Assistance requested to evaluate the need for daily living aids. ADL assistance was offered to 2–3 patients per week	No ADL assistance	Evaluation of the need for daily living and occupational therapy (ADL) was offered to all patients thought able to benefit from it (five patients per week).
Nutritional therapy	Assistance requested to develop nutrition plans (five minutes per patient)	No support from dieticians	Attending conferences, assessing patients’ nutritional status, and developing nutrition plans.
Discharge planning	Early discharge planning. Report sent to the municipality for all patients with established contact. Video conference when major changes were needed.	Early discharge planning. Report was sent to the municipality for all patients with established contact. Video conference when major changes were needed.	Early discharge planning. Report was sent to the municipality for all patients with established contact. Video conference when major changes were needed.
Staff training	No specific training	No specific training	A 6 × 3-h course for carers in orthogeriatric care and medical knowledge including sessions on preventing, detecting, and treating various medical complications.

### Study design and participants

A prospective observational cohort study with a retrospective (historical) control was designed.

The participants were all patients aged 65 years or older admitted to the orthogeriatric unit with a fragility fracture during two study periods: September 1, 2013 to January 31, 2014 (the historical cohort) and between September 1, 2014 and August 31, 2015 (the orthogeriatric cohort).

Patients were excluded if the fracture was cancer-related or caused by high-energy trauma, if the patient was operated on at another hospital, treated conservatively with no operation, or had been readmitted within the last month due to fracture-related complications.

Fragility fractures were diagnosed by the orthopaedic surgeon in the Emergency room by the definition: fractures occurring after minimal trauma, such as falling from a standing height or less, or after no identifiable trauma [26]. The fragility fractures included were hip fractures, clinical vertebral fractures, and appendicular fractures, with the exception of patients with fractures of the skull, face, fingers, hands, feet, toes, or kneecaps, as these fractures were not defined as fragility fractures. Hip fractures were identified as DS72, vertebral fractures as DS22 and DS32, and appendicular fractures as DS42, DS52, DS821–9, using codes from the International Classification of Diseases, version 10 (ICD10).

### Outcome variables

The primary outcome of interest in our study was postoperative complications, defined as the proportion of patients with at least one of the following events; medical complications (cardiac, cerebral, thrombo-embolic, pulmonary, gastro-intestinal complications, urinary tract infection, delirium, pressure ulcer and subsequent fracture – new fractures during admission unrelated to the first fracture) or surgical complications (surgical site infections and surgical complications in terms of luxation) occurring at any time between operation and discharge, as recommended by Liem et al. [23]. Adverse drug reactions (ADR) and renal complications - i.e., transient or lasting increases in serum creatinine levels—were not included, as these cases were inappropriately defined and not systematically assessed. Additionally, the number of complications per patient was assessed numerically (0, 1 or more).

We differentiated between preoperative and postoperative complications by the time the complication was recognized. A medical complication was defined as a new medical condition or a destabilization of a previously stable illness.

Neither the Confusion Assessment Method (CAM) nor the guideline-specific initiatives of delirium management were systematically employed in the prior orthopaedic organization nor implemented during our investigation. Therefore, as both criteria were not met delirium was defined as the state of a patient described being delirious in the medical record and receiving haloperidol treatment as recommended in the local guideline.

The secondary outcome of interest was readmission—defined as any admission within 30 days from discharge.

### Patient and admission-related characteristics

Patient characteristics included age, gender, marital status, BMI, place of residence, use of walking aid (yes/no), mobility before fracture using a mobility score validated for hip fracture patient (the Cumulated Ambulation Score (CAS) [27]; only collected for hip fracture patients), and comorbidity using Charlson’s Comorbidity Index (CCI). Comorbidity data were weighted according to the Charlson protocol and an index score was calculated for each patient [28].

Characteristics related to admission and operation included type of fracture, number of drugs at the time of admission, polypharmacy (defined as 5 or more different medications at admission), and the American Society of Anaesthesiologists Physical Status (ASA score) - a grading system from 1 to 6 used to evaluate patients’ physical state before choosing an anaesthetic. Furthermore, we assessed preoperative complications, patient ambulation within 24 h after operation (yes/no), pain score on the second after the operation, mobility at discharge using CAS, time to surgery (TTS), and length of stay (LOS). Time to surgery (TTS) was defined as time (hours) from recorded admission time to the time anaesthesia began, and length of stay (LOS) was defined as the number of hours for which the patient was hospitalized.

### Data sources

Data on age, gender, place of fall, type of fracture, TTS, and LOS were obtained from the patient administrative system, and data on ASA was sourced from the Danish Anaesthesia Database. Comorbidity data and data on readmission were collected from a national registry using diagnoses listed from all hospital discharges for a period from 1994 until 1 month after current admission [29]. All remaining variables were collected from medical records.

### Statistical analysis

The measurements of postoperative complications and readmission are expressed as proportions. Furthermore, postoperative complications and readmissions are examined using a binary logistic regression model on the individual patient level; adjusting for age and gender, and CCI or LOS, respectively. Subsequently regressions are made solely for hip fractures.

Numeric patient and admission-related characteristics are expressed as medians (quartiles) or mean values (±SDs) when appropriate; the unpaired Student’s *t*-test or the Mann–Whitney *U*-test is used depending on data distribution. When assessing categorical variables, we used proportions and the chi-squared test.

A two-sample comparison of proportions with a 1:2 patient ratio was chosen to generate more power to detect postoperative complications after implementing the intervention. On the basis of a significant 15% difference in postoperative complications in hip fracture patients assigned to multidisciplinary geriatric intervention versus the traditional orthopaedic care [8] and in the absence of results generated in study populations characterised by fragility fractures in general, a sample size of 183 (in the first period) and 366 (in the second period) hip fracture patients is necessary to detect a 15% decrease in postoperative complications in the intervention group, setting α at 0.05 and β at 0.9. In addition, patients with additional fragility fractures were concurrently included.

All analyses were performed using Stata 13 software (Stata Statistical Software: Release 13, 2013, College Station, TX).

## Results

### Patient characteristics and characteristics related to admission

**Fig. 1:**
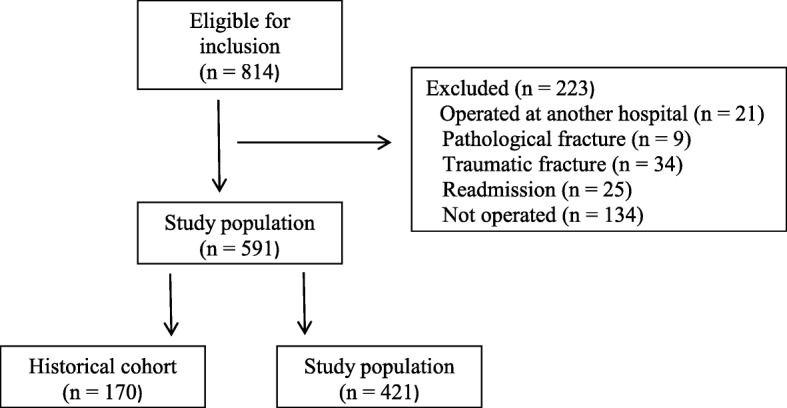
Flowchart

We identified 814 patients eligible for inclusion. In total, 223 patients were excluded on the basis of the exclusion criteria or not having been operated on. We thus included 591 patients: 170 in the historical cohort and 421 in the orthogeriatric cohort (Fig. 1).

The mean age was 80.2 years (SD 8.4); 77.5% were women, 40.9% were cohabiting, and 79.5% were living in their own home. The mean BMI was 23.7 (SD 4.4), and 46.9% of the participants used walking aids. Polypharmacy occurred in 61.9% of the patients. A total of 66.8% of the participants were admitted due to hip fracture (DS72), 6.4% due to humerus fracture (DS42), 21.2% due to radius fracture (DS52), and 5.6% due to tibia fracture (DS821–9). At admission, the patients had a mean CCI of 1.6 (SD 2.0), and the most prevalent ASA score was 3 (46.5%); the hip fracture patients at admission had a mean CAS of 4.8 (SD 1.8). Preoperative complications i.e. complications diagnosed from admission until entering the operating room were found in 9.4% of participants; including 2.4% with pneumonia and in 4.4% with urinary tract infection. In total, 88.3% of the hip fracture patients were ambulated within 24 h. The mean time to surgery was 26.2 h (SD 25.33) and the mean length of stay was 153.9 h (SD 92.1).

The two cohorts were significantly different with regard to marital status, as a larger proportion in the historical cohort was living alone (67.3% vs 55.6%, *p* = 0.01). Furthermore, a significantly larger proportion of patients in the orthogeriatric cohort used walking aids at time of admission (34.7% vs 51.8%, *p* = 0.001) and had a better CAS before admission (median 6 (2–6) vs 6 (5–6), *p* <  0.001) and at discharge (median 2 (1–5) vs 3 (2–5), *p* = 0.007); CAS thus decreased from admission to discharge in both cohorts.

The overall time to surgery was longer in the orthogeriatric cohort (18.2 vs 20.5 h, *p* = 0.01). In both cohorts, time to surgery was 2–5 h longer in patients with appendicular fractures compared to those with hip fractures. The length of stay remained unchanged in the total orthogeriatric cohort (141.6 vs 145.7 h, *p* = 0.14); yet was significantly prolonged among those with appendicular fractures (64.1 vs. 91.0 h, *p* = 0.002) (Table 2).

**Table 2:** Patient characteristics and characteristics related to admission

Patient characteristics	Historical cohort (*n* = 170)	Orthogeriatric cohort (*n* = 421)	*P*-value
Age, median (p25-p75)	81 (73–81)	80 (73–80)	0.31
Female	78.2	77.2	0.78
Marital status, % (*n* = 540)			
Widow/living alone	67.3	55.6	**0.01**
Married/cohabiting	32.7	44.4	
Place of residence, % (*n* = 560)			
Nursing home	13.2	15.3	0.40
Sheltered housing	7.8	5.1	
Own home	79.0	79.6	
BMI, median (p25-p75) (*n* = 439)	23 (20–26)	23.2 (21–26)	0.44
Prefracture health status			
Use of walking aid, % (*n* = 437)	34.7	51.8	**0.001**
CAS, median (p25-p75) (*n* = 330, hip fracture)	6 (2–6)	6 (5–6)	**< 0.001**
Charlson Comorbidity Index, median (p25-p75)	1 (0–2)	1 (0–2)	0.05
Characteristics related to admission			
Type of fracture, %			
Hip fracture (DS72), (n = 395)	68.8	66.0	0.51
Appendicular fracture (n = 196)	31.2	34.0	
Clavicular, humeral (DS42)	5.9	6.5	
Radius, ulna, colles (DS52)	25.3	19.5	
Tibia, malleolus (DS82)	NA	7.8	
Medication at admission			
Polypharmacy (> 5 drugs) %	62.9	61.5	0.75
Medication at admission, median (p25-p75)	6 (3–9)	6 (3–9)	0.64
ASA-score, median (p25-p75) (*n* = 585)	3 (2–3)	3 (2–3)	0.50
Score 1, %	7.8	5.3	0.32
Score 2, %	39.5	42.1	
Score 3, %	48.5	44.5	
Score 4, %	4.2	7.9	
Score 6, %	NA	0.2	
Preoperative complications, % (*n* = 587)	8.2	9.8	0.55
Ambulation within 24 h after surgery, %			
Hip fracture, (*n* = 360)	91.3	87.2	0.27
Pain on day 2 after surgery, % (*n* = 322)			
NRS 0	8.0	10.8	0.30
NRS 1–3	35.0	42.8	
NRS 4–6	49.0	38.3	
NRS 7–10	8.0	8.1	
Discharge CAS, median (p25-p75) (*n* = 339, hip fracture)	2 (1–5)	3 (2–5)	**0.007**
Time to surgery, median (p25-p75)	18.2 (11.4–25.2)	20.5 (13.0–31.0)	**0.01**
Hip fracture	17.8 (9.8–23.2)	19.4 (12.4–25.3)	0.06
Appendicular fracture	20.3 (14.1–36.0)	24.7 (13.7–46.8)	0.12
Length of stay, hours median (p25-p75)	141.6 (66.7–201.3)	145.7 (82.0–212.4)	0.14
Hip fracture	167.3 (126.8–225.3)	168.0 (119.2–231.5)	0.80
Appendicular fracture	64.1 (41.9–96.1)	91.0 (51.8–157.8)	**0.002**

* bold indicates statistical level < 0.05

### Postoperative complications

We found no significant differences between the cohorts with regard to the proportion of participants experiencing complications (24.5% versus 28.3%, *p* = 0.36) (Table 3). Hence, medical complications occurred in 23.3% versus 27.1% of the study participants, respectively (p = 0.36). When adjusted for age, gender, and comorbidity, the odds of postoperative complication increased numerically in the orthogeriatric cohort, compared to the historic cohort, from 1.21 (CI: 0.80 to 1.83) to 1.35 (CI: 0.88 to 2.08); the results nonetheless remained insignificant (*p* = 0.17).

**Table 3:** Analysis of postoperative complications between historical and orthogeriatric cohort

	Historical Cohort (*n* = 170)	Orthogeriatric cohort (*n* = 421)	Odds ratio of postoperative complications in historical versus orthogeriatric cohort			
	%	%	OR (95% CI)	*P*-value*	Adjusted OR (95% CI)	*P*-value*
Overall complications (*n* = 577)	24.5	28.3	1.21 (0.80–1.18)	0,36	1.35 (0.88–2.08)	0.17
Medical complications (*n* = 577)	23.3	27.1	1.22 (0.80–1.85)	0.36	1.36 (0.88–2.10)	0.17
Delirium (*n* = 585)	3.0	5.5	1.90 (0.71–5.10)	0.20	2.07 (0.76–5.67)	0.16
Urinary tract infection (*n* = 585)	11.9	11.0	0.92 (0.52–1.60	0.76	0.98 (0.56–1.74)	0.96
Pulmonary complications (*n* = 585)	8.3	14.1	1.81 (0.98–3.34)	0.06	2.00 (1.06–3.78)	**0.03**
Pneumonia	7.1	13.7	2.06 (1.07–3.94)	**0.03**	2.33 (1.18–4.59)	**0.01**
Exacerbation of COPD^	1.2	1.2	1.01 (0.19–5.24)	0.99	0.97 (0.18–5.28)	0.97
Cardiac complications	4.8	4.3	0.90 (0.38–2.12)	0.81	1.02 (0.42–2.47)	0.96
Arrhythmia	3.6	4.1	1.15 (0.44–2.96)	0.78	1.26 (0.48–3.32)	0.64
Congestive heart failure	0	0.7	1		1	
Myocardial infarction	1.2	0.7	0.60 (0.99–3.63)	0.58	0.75 (0.12–4.74)	0.76
Cerebral complications	0	0.2	1		1	
Thromboembolic complications	1.2	0.5	0.40 (0.56–2.86)	0.36	0.34 (0.05–2.49)	0.29
Deep vein thrombosis	0.6	0.5	0.80 (0.72–8.94)	0.86	0.69 (0.61–7.80)	0.77
Pulmonary embolism	0.6	0.24	0.40 (0.25–6.45)	0.52	0.33 (0.02–5.61)	0.44
Gastrointestinal (GI) complications	0	1.9	1		1	
Ileus	0	0.5	1		1	
Gastrointestinal bleeding	0	1.7	1		1	
Pressure ulcer (*n* = 577)	0	0	1		1	
Subsequent fracture	0.6	0.5	0.80 (0.07–8.94)	0.86	0.82 (0.07–9.27)	0.87
Surgical complication (*n* = 585)	1.2	1.7	1.42 (0.29–6.89)	0.67	1.47 (0.30–7.21)	0.64
Surgical site infection	0.6	1.2	2.03 (0.24–17.48)	0.52	2.36 (0.27–20.7)	0.44
Surgical complication	1.2	0.7	0.61 (0.10–3.65)	0.58	0.54 (0.08–3.37)	0.51
Complications per patient (*n* = 577)						
= 0	75.4	71.7	1		1	
= 1	19.8	20.2	1.08 (0.68–1.70)	0.75	1.21 (0.76–1.93)	0.43
≥ 2	4.8	8.1	1.77 (0.79–3.93)	0.16	1.99 (0.87–4.54)	0.10

^*COPD* Chronic Obstructive Pulmonary Disease

*p values were calculated using logistic regression, adjusted for age, gender and CCI

* bold indicates statistical level < 0.05

The most common medical complications were urinary tract infection and pneumonia; pneumonia was more common in the orthogeriatric cohort (*p* = 0.03). A total of 72.8% of all patients did not experience complications, with 20.1% experiencing one and 7.1% two complications; there were no differences between the two cohorts (Table 3).

When comparing postoperative complications among hip fracture patients in the two cohorts, differences remained insignificant (33.9% vs. 37.7%, *p* = 0.48).

Once regressions were performed between patients with hip fractures and those with appendicular fractures, with both cohorts collapsed, the odds of having postoperative complications as a hip fracture patient were 5.99 times greater than for patients with appendicular fracture (*p* <  0.001); by adjusting for age, gender, and CCI, the odds ratio was reduced to 4.45 (*p* <  0.001). Patients with hip fractures were older (82 vs 75 years, *p* <  0.002), had a higher CCI (1 (0–2) vs 1 (0–3), *p* <  0.002), and more were men (27.9% vs 11.7%, *p* <  0.001) than in the case of patients with appendicular fractures.

Patients with complications were more likely to be older (84.0 vs 78.5, *p* <  0.001), male (29.9 vs 20.0, *p* = 0.02), with a hip fracture (89.2 vs 57.9, *p* <  0.001), with a higher CCI (1 vs 1, *p* = 0.02), and to have an ASA score of 3–4 (63.9 vs 48.3, *p* = 0.004) compared to patients without complications (Table 4). Additionally, patients with complications had longer stays in hospital (206.6 vs 117.5 h, *p* <  0.001) (Table 4).

**Table 4:** Patient characteristics without and with postoperative complications

	Patients without postoperative complications (*n* = 420)	Patients with postoperative complications (*n* = 157)	*P*-value
Age, median (p25-p75)	78.5 (72–85)	84 (79–89)	**< 0.001**
Male, %	20.0	29.9	**0.02**
Type of fracture, %			
Hip fracture (DS72)	57.9	89.2	**< 0.001**
Appendicular fracture	42.1	10.8	
Prefracture health status			
Use of walking aid, %	41.4	59.9	**< 0.001**
Charlson Comorbidity Index, median (p25-p75**)**	1 (0–2)	1 (0–3)	**0.007**
ASA-score, median (p25-p75) (*n* = 571)	2 (2–3)	3 (2–3)	**< 0.001**
Score 1–2, %	51.4	36.1	**0.004**
Score 3–4, %	48.3	63.9	
Score 5–6, %	0.3	0	
Length of stay, hours median (p25-p75)	117.5 (65.4–173.7)	206.6 (147.3–265.3)	**< 0.001**
Readmission	9.5	22.3	**< 0.001**

* bold indicates statistical level < 0.05

Compared to patients admitted with other fragility fractures, hip fracture patients had a higher incidence of at least one preoperative complication (17.1% vs 5.1%, *p* < 0.001) and of having at least one postoperative complication (36.5% vs 8.8%, *p* < 0.001). Hip fracture patients had a higher risk of experiencing delirium, urinary tract infection, sepsis and pneumonia after surgery than patients with appendicular fractures (*p* < 0.001). In patients with an appendicular fracture, postoperative complications were more frequent in those admitted with a tibial fracture (21.2%), than those who experienced a humerus and radius fracture (11.9% vs 4.0%), respectively (both *p* < 0.001).

Furthermore, we found that polypharmacy was associated with a higher readmission rate in hip fracture patients (*p* = 0.04) and that a CCI of 2 or more was associated with a higher readmission rate in patients admitted with an appendicular fracture (*p* = 0.04) (Table 5).

**Table 5:** Odds ratio of readmission and postoperative complications by CCI and polypharmacy in patients with hip or appendicular fracture

	*n* (%)	Odds ratio of postoperative complications by CCI and polypharmacy in patients with hip or appendicular fracture		Odds ratio of readmission by CCI and polypharmacy in patients with hip or appendicular fracture	
		OR (95% CI)	*P*-value*	OR (95% CI)	*P*-value*
Hip (*n* = 395)					
Polypharmacy	267 (67.6)	0.80 (0.51–1.24)	0.32	1.92 (1.02–3.60)	**0.04**
Charlson Comorbidity Index					
0	124 (31.4)	1		1	
1	89 (22.5)	1.12 (/0.66–1.90)	0.68	1.29 (0.62–2.70)	0.50
≥2	182 (46.1)	1.45 (0.93–2.28)	0.10	1.21 (0.62–2.70)	0.56
Appendicular fracture (*n* = 196)					
Polypharmacy	99 (50.5)	0.84 (0.31–2.28)	0.73	2.38 (0.60–9.5)	0.22
Charlson Comorbidity Index					
0	91 (46.4)	1		1	
1	51 (26.0)	1.52 (0.44–5.26)	0.51	1.8 (0.25–13.30)	0.56
≥ 2	54 (27.6)	1.79 (0.54–5.85)	0.34	5.6 (1.10–26.63)	**0.04**

* bold indicates statistical level < 0.05

### Readmission

Taking into account the readmissions within 30 days after discharge, we found no significant difference in the historical cohort vs the orthogeriatric cohort (14.1% vs 12.1%, p = 0.5) (Table 6). When adjusted for age, gender, and LOS, the odds of readmission decreased numerically in the orthogeriatric cohort, compared to the historic cohort, from 0.84 (CI 0.50–1.41) to 0.80 (CI 0.46–1.38), but the results nonetheless remained insignificant (*p* = 0.42).

Furthermore, we found no significant difference when analysing readmissions for hip fracture patients exclusively (17.9% vs 15.8%, *p* = 0.6) (Table 6).

In the orthogeriatric cohort patients with complications had 2.1 (CI 1.17–3.90) higher odds of readmission within 30 days than those without complications (*p* = 0.013) whereas in the historic cohort, patients with complications had 4.8 (1.97–11.97) higher odds of readmission within 30 days than those without complications (*p* = 0.001).

With both cohorts collapsed, 65 out of 75 readmissions occurred in patients with hip fractures (Table 6). Diagnosis at readmission included respiratory difficulties (*n* = 13; pneumonia and COPD), other causes (*n* = 12; fatigue, dizziness, pain, and syncope), complications to surgery (surgical (*n* = 4), haemorrhagic (*n* = 4), and infection (*n* = 2)), circulatory problems (*n* = 9; myocardial infarction, tachycardia, heart failure, and apoplexy), infection (*n* = 8; sepsis and bacterial infection), and anaemia (n = 4).

**Table 6:** Analysis of readmissions between historical and orthogeriatric cohort

	Historical Cohort (*n* = 170)	Ortho geriatric cohort (*n* = 421)	Odds ratio of readmissions in historical versus orthogeriatric cohort			
	%	%	OR (95% CI)	*P*-value*	Adjusted OR (95% CI)	*P*-value*
Readmission (*n* = 75)	14.1	12.1	0.84 (0.50–1.41)	0.51	0.80 (0.46–1.38)	0.42
Hip fracture (*n* = 65)	17.9	15.8	0.86 (0.49–1.52)	0.60	0.85 (0.47–1.54)	0.60

*p values were calculated using logistic regression, adjusted for age, gender and LOS

In the collapsed study population, patients with complications during admission had a higher risk of readmission within 30 days than did patients who had not experienced complications during admission (22.3% vs 9.5%; *p* < 0.001) (Table 4). The odds of being readmitted among those who experienced a postoperative complication was 2.72 times greater (CI 1.65–4.48) than in patients not experiencing postoperative complications.

## Discussion

This study investigated the effect of an orthogeriatric intervention in patients of 65 years or older who were admitted with a fragility fracture and operated on at a Danish regional hospital.

We found no significant differences in the proportion of patients with postoperative complications when comparing orthogeriatric care to the traditional care. A slight increase was nevertheless found in the orthogeriatric cohort, which could be explained by the greater focus of geriatricians on diagnosing and treating medical diseases, as mentioned by other researchers [13].

Our postoperative complication rate of 33.9% in the historical hip fracture cohort was distinctly lower than those reported in other studies [4, 6–9, 13]; in other studies, complication rates in units without orthogeriatric care varied from 46.3 to 71%. However, a wide range of different complications have been reported, as there has been no consensus regarding the definition, classification, or assessment of complications [30]. On closer examination, postoperative complications were measured differently regarding 1) their number (varying from 8 to 16), 2) whether they were solely medical, or included all complications, and 3) their type; this makes it challenging to compare results in terms of proportions.

Finding no significant differences in postoperative complications between the two cohorts, we hypothesized that the effect of orthogeriatric care would appear as a decrease in the proportion of long-term readmissions (30 days). However, we found no differences in readmission rates when comparing orthogeriatric care to traditional care. The literature seems to reveal inconsistency in the study of readmission rates; two prospective observational studies with retrospective (historical) control cohorts found decreases in such rates [4, 5], while several studies found no differences [6, 9, 31, 32]; the time lapse before assessing readmission rates varied from 30 days [5, 6, 32] to 12 months [31]. Valid comparison of readmission rates was also hindered by variation as to the data sources. Some studies thus included readmission data from all wards in the hospital while other studies included only the orthogeriatric ward.

The main reason for readmission within 30 days was medical complications. One study of hip fracture patients admitted to an orthopaedic ward and consulted immediately by a geriatrician differentiated among causes of readmissions within 30 days, showing that 1.8% of the complications were surgical and 8.5% were medical [33]. This distribution supports our findings on medical complications being the main cause.

Furthermore, we found an association between postoperative complications and readmission within 30 days. No similar association was reported in earlier studies investigating both postoperative complications and readmissions. Some studies report a decrease in postoperative complications comparing traditional care to orthogeriatric care, yet no difference in readmission rates were found [6, 9]; other studies [4, 5] found a significant decrease in both postoperative complications and readmissions. Thus, the overall changes in postoperative complications are not consistently associated with variations in readmission rates.

With both cohorts collapsed, we also found the risk of postoperative complications to be 4.5 times higher in patients admitted due to a hip fracture compared to those with an appendicular fracture. To our knowledge, no other researchers have reported similar results. The higher risk of complications in hip fracture patients may be explained by longer duration of surgery, more frequent risk of postoperative immobilization and higher proportion being frail than patients with other fragility fractures.

Furthermore, we found polypharmacy in hip fracture patients to be associated with readmission. Although it is unclear why polypharmacy increases readmission rates in patients with hip fractures, it could be speculated that the need for readmission may arise due to treatment-related side effects or that polypharmacy is a marker of comorbidities. Moreover, with both cohorts collapsed, we found that patients with postoperative complications were most likely to be older, male, with more comorbidities, and to have ASA scores of 3–4 in comparison with patients without complications—corroborating other studies showing that postoperative complications are more common in patients who are older [6, 25], male [25], with ASA scores of 3–4 [34, 35], and with high comorbidity levels [3, 25, 36].

Several studies support our findings of pneumonia and urinary tract infection as the most common complications when admitted with hip fractures [5, 7, 9, 20, 25, 34, 35]. Additionally, delirium has been reported as a common complication, though the proportion of patients with delirium varied from 5.9 to 39%, respectively [4, 5, 7]. The incidence of delirium was lower in our investigation than reported in previous studies. Due to absence of consistent assessments of delirium in both cohorts, delirium was based on the clinical diagnosis as determined and documented by clinical staff and use of haloperidol. As per a local guideline haloperidol was prescribed in a limited number of circumstances; therefore, the incidence of delirium was underestimated in our investigation.

We additionally found a significantly prolonged length of stay among patients with postoperative complications, compared to patients without complications. These results were also found in other studies [24, 37].

Patients admitted with fragility fractures form a heterogeneous group with many different types of fractures, different comorbidity levels, and different levels of functional ability. We have provided restricted analyses for hip fracture patients in order to relate our results to a well-known group of patients and to be able to compare results between patients with hip fractures and those with appendicular fractures. Our results did not change when we analysed the subgroup of hip fracture patients.

Since 1999, all orthopaedic surgery departments in Denmark have worked according to a national Reference Programme for hip patients [38]. The programme includes recommendations on time to surgery, early mobilization, clarifying ambulation status before fracture and at discharge, nutritional status, initiating in-hospital osteoporosis treatment, and fall prevention, in order to increase quality of care. Comparing the results of our study to the quality of care recommendations for hip fracture patients in Denmark, the mean time to surgery was below the recommended 24 h, and ambulation within 24 h of the operation occurred in about 90% of all hip patients in our investigation, indicating a good quality of care in our setting, both before and after implementing orthogeriatric care.

Patient characteristics in the two cohorts were comparable, although patients from the orthogeriatric cohort had a significantly higher CAS, higher use of walking aids, and were more likely to be cohabiting.

### Strengths and limitations

In examining postoperative complications, we did not distinguish between minor and major complications, and neither did we take into account the severity of complications. However, we have categorized complications as preoperative or postoperative and accounted for complications, as recommended [23]. However, ADR and renal complications are underrepresented, as these cases were inappropriately defined and not systematically documented in the records.

Calculating a sample size on the basis of a 2005 article [8] that showed a 15% decrease in postoperative complications may have overestimated the potential effects of orthogeriatric care. Furthermore, detecting a significant change of 15% from our initially low complication rate of 24.5% was next to impossible.

## Conclusion

In older patients admitted with and surgically treated for fragility fractures, our model of orthogeriatric care showed no significant differences regarding postoperative complications or readmissions, compared to traditional care. We did, however, find higher risk of postoperative complications among patients admitted with a hip fracture compared to other fragility fractures. Additionally, our study reveals an increased risk of being readmitted within 30 day for patients with postoperative complications.

Our results contribute to the knowledge of the impact of orthogeriatric care in older patients with various types of fragility fractures.

Further studies on specific subgroups of fractures, as well studies on other relevant outcomes such as mortality, are recommended.

## Data Availability

The datasets generated and analysed during the current study are not publicly available due to Danish legislation. However, the corresponding author will be happy to answer any question about data.

## Abbreviations

ADR: Adverse drug reactions
ASA: The American Society of Anaesthesiologists Physical Status
CAM: The Confusion Assessment Method
CAS: The Cumulated Ambulation Score
CCI: Charlson’s Comorbidity Index
ICD: The International Classification of Diseases
ICU: Intensive care unit
LOS: Length of stay
TTS: Time to surgery

## References

[1] Braithwaite RS, Col NF, Wong JB (2003). Estimating hip fracture morbidity, mortality and costs. J Am Geriatr Soc.

[2] Cooper C, Melton LJ (1992). Epidemiology of osteoporosis. Trends Endocrinol Metab.

[3] Kammerlander C, Roth T, Friedman SM, Suhm N, Luger TJ, Kammerlander-Knauer U (2010). Ortho-geriatric service--a literature review comparing different models. Osteoporos Int.

[4] Fisher AA, Davis MW, Rubenach SE, Sivakumaran S, Smith PN, Budge MM (2006). Outcomes for older patients with hip fractures: the impact of orthopedic and geriatric medicine cocare. J Orthop Trauma.

[5] Folbert EC, Smit RS, van der Velde D, Regtuijt EM, Klaren MH, Hegeman JH (2012). Geriatric fracture center: a multidisciplinary treatment approach for older patients with a hip fracture improved quality of clinical care and short-term treatment outcomes. Geriatr Orthop Surg Rehabil.

[6] Friedman SM, Mendelson DA, Bingham KW, Kates SL (2009). Impact of a comanaged geriatric fracture center on short-term hip fracture outcomes. Arch Intern Med.

[7] Khasraghi FA, Christmas C, Lee EJ, Mears SC, Wenz JF (2005). Effectiveness of a multidisciplinary team approach to hip fracture management. J Surg Orthop Adv.

[8] Vidan M, Serra JA, Moreno C, Riquelme G, Ortiz J (2005). Efficacy of a comprehensive geriatric intervention in older patients hospitalized for hip fracture: a randomized, controlled trial. J Am Geriatr Soc.

[9] Dy CJ, Dossous PM, Ton QV, Hollenberg JP, Lorich DG, Lane JM (2012). The medical orthopaedic trauma service: an innovative multidisciplinary team model that decreases in-hospital complications in patients with hip fractures. J Orthop Trauma.

[10] Katrancha ED, Zipf J, Abrahams N, Schroeder R (2017). Retrospective evaluation of the impact of a geriatric trauma institute on fragility hip fracture patient outcomes. Orthop Nurs.

[11] Duaso E, Formiga F, Marimon P, Sandiumenge M, Salgado MT, Murga V (2018). Advantages of care for patients with hip fractures in the acute geriatric unit: hip study Anoia. Geriatr Gerontol Int.

[12] Barone A, Giusti A, Pizzonia M, Razzano M, Palummeri E, Pioli G (2006). A comprehensive geriatric intervention reduces short- and long-term mortality in older people with hip fracture. J Am Geriatr Soc.

[13] Leung AH, Lam TP, Cheung WH, Chan T, Sze PC, Lau T (2011). An orthogeriatric collaborative intervention program for fragility fractures: a retrospective cohort study. J Trauma.

[14] Zeltzer J, Mitchell RJ, Toson B, Harris IA, Ahmad L, Close J (2014). Orthogeriatric services associated with lower 30-day mortality for older patients who undergo surgery for hip fracture. Med J Aust.

[15] Baroni M, Serra R, Boccardi V, Ercolani S, Zengarini E, Casucci P (2019). The orthogeriatric comanagement improves clinical outcomes of hip fracture in older adults. Osteoporos Int.

[16] Forni S, Pieralli F, Sergi A, Lorini C, Bonaccorsi G, Vannucci A (2016). Mortality after hip fracture in the elderly: the role of a multidisciplinary approach and time to surgery in a retrospective observational study on 23,973 patients. Arch Gerontol Geriatr.

[17] Lips, P. (1997). "epidemiology and predictors of fractures associated with osteoporosis." am J med **103**(2a): 3S-8S; discussion 8S-11S. 10.1016/s0002-9343(97)90021-8 9302892

[18] Aw D, Sahota O (2014). Orthogeriatrics moving forward. Age Ageing.

[19] Sabharwal S, Wilson H (2015). Orthogeriatrics in the management of frail older patients with a fragility fracture. Osteoporos Int.

[20] Kammerlander C, Gosch M, Blauth M, Lechleitner M, Luger TJ, Roth T (2011). The Tyrolean geriatric fracture center: an orthogeriatric co-management model. Z Gerontol Geriatr.

[21] Chong C, Christou J, Fitzpatrick K, Wee R, Lim WK (2008). Description of an orthopedic-geriatric model of care in Australia with 3 years data. Geriatr Gerontol Int.

[22] Adams AL, Schiff MA, Koepsell TD, Rivara FP, Leroux BG, Becker TM (2010). Physician consultation, multidisciplinary care, and 1-year mortality in Medicare recipients hospitalized with hip and lower extremity injuries. J Am Geriatr Soc.

[23] Liem IS, Kammerlander C, Suhm N, Blauth M, Roth T, Gosch M (2013). Identifying a standard set of outcome parameters for the evaluation of orthogeriatric co-management for hip fractures. Injury.

[24] Khasraghi FA, Lee EJ, Christmas C, Wenz JF (2003). The economic impact of medical complications in geriatric patients with hip fracture. Orthopedics.

[25] Roche JJ, Wenn RT, Sahota O, Moran CG (2005). Effect of comorbidities and postoperative complications on mortality after hip fracture in elderly people: prospective observational cohort study. Bmj.

[26] Brown JP, Josse RG (2002). 2002 clinical practice guidelines for the diagnosis and management of osteoporosis in Canada. Cmaj.

[27] Foss NB, Kristensen MT, Kehlet H (2006). Prediction of postoperative morbidity, mortality and rehabilitation in hip fracture patients: the cumulated ambulation score. Clin Rehabil.

[28] Charlson ME, Pompei P, Ales KL, MacKenzie CR (1987). A new method of classifying prognostic comorbidity in longitudinal studies: development and validation. J Chronic Dis.

[29] Thygesen SK, Christiansen CF, Christensen S, Lash TL, Sorensen HT (2011). The predictive value of ICD-10 diagnostic coding used to assess Charlson comorbidity index conditions in the population-based Danish National Registry of patients. BMC Med Res Methodol.

[30] Liem IS, Kammerlander C, Suhm N, Kates SL, Blauth M (2014). Literature review of outcome parameters used in studies of geriatric fracture centers. Arch Orthop Trauma Surg.

[31] Deschodt M, Braes T, Broos P, Sermon A, Boonen S, Flamaing J (2011). Effect of an inpatient geriatric consultation team on functional outcome, mortality, institutionalization, and readmission rate in older adults with hip fracture: a controlled trial. J Am Geriatr Soc.

[32] Stenvall M, Olofsson B, Nyberg L, Lundstrom M, Gustafson Y (2007). Improved performance in activities of daily living and mobility after a multidisciplinary postoperative rehabilitation in older people with femoral neck fracture: a randomized controlled trial with 1-year follow-up. J Rehabil Med.

[33] Kates SL, Blake D, Bingham KW, Kates OS, Mendelson DA, Friedman SM (2010). Comparison of an organized geriatric fracture program to United States government data. Geriatr Orthop Surg Rehabil.

[34] Kua J, Ramason R, Rajamoney G, Chong MS (2016). Which frailty measure is a good predictor of early post-operative complications in elderly hip fracture patients?. Arch Orthop Trauma Surg.

[35] Pedersen SJ, Borgbjerg FM, Schousboe B, Pedersen BD, Jorgensen HL, Duus BR (2008). A comprehensive hip fracture program reduces complication rates and mortality. J Am Geriatr Soc.

[36] Friedman SM, Mendelson DA, Kates SL, McCann RM (2008). Geriatric co-management of proximal femur fractures: total quality management and protocol-driven care result in better outcomes for a frail patient population. J Am Geriatr Soc.

[37] Biber R, Singler K, Curschmann-Horter M, Wicklein S, Sieber C, Bail HJ (2013). Implementation of a co-managed geriatric fracture center reduces hospital stay and time-to-operation in elderly femoral neck fracture patients. Arch Orthop Trauma Surg.

[38] [Referenceprogram for patienter med hoftebrud] Reference program for patients with hip fracture (2008) Denmark. ; http://www.ortopaedi.dk/fileadmin/Guidelines/Referenceprogrammer/Referenceprogram_for_patienter_med_hoftebrud2008.pdf . .

